# International Congress of Forensic Medicine

**Published:** 1897-06

**Authors:** 


### International Congress of Forensic Medicine.

The "Société de Médecine légale de Belgique" is organising an International Congress on this important subject. It is to be held in Brussels from August 2nd to the 7th, and will concern itself with the following topics: Bacteriology and Toxicology; Legislation; Forensic Medicine; Mental Medicine. The cost of membership is 20 francs. Further information can be obtained from the Secretary-General, Dr. Camille Moreau, Charleroi. The fact that the Congress is to be held during the time the Brussels Exhibition will be open may be a special inducement to those who are thinking of attending.

